# A Medical Jest

**Published:** 1909-08

**Authors:** 


### A Medical Jest.

There's naught to make the sufferer grin,
In abscess, boil or tumor;
Although physicians find therein
Considerable humor.

—*The Doctor.*

